# A novel immunohistochemical score to predict early mortality in acute myeloid leukemia patients based on indoleamine 2,3 dioxygenase expression

**DOI:** 10.1038/s41598-017-12940-0

**Published:** 2017-10-16

**Authors:** Abhishek Mangaonkar, Ashis Kumar Mondal, Sadanand Fulzule, Chetan Pundkar, Eun Jeong Park, Anand Jillella, Vamsi Kota, Hongyan Xu, Natasha M. Savage, Huidong Shi, David Munn, Ravindra Kolhe

**Affiliations:** 1 Department of Hematology and Medical Oncology, Mayo Clinic, Rochester, USA; 2 Department of Pathology, Medical College of Georgia at Augusta University, Augusta, USA; 3 Department of Orthopedics, Medical College of Georgia at Augusta University, Augusta, USA; 4 Department of Hematology and Medical Oncology, Emory University, Atlanta, USA; 5 Department of Population Health Sciences, Medical College of Georgia at Augusta University, Augusta, USA; 6 Georgia Cancer Center, Medical College of Georgia at Augusta University, Augusta, USA; 7 Department of Biochemistry and Molecular Biology, Medical College of Georgia at Augusta University, Augusta, USA; 8 Department of Pediatrics, Medical College of Georgia at Augusta University, Augusta, USA

## Abstract

Indoleamine 2,3 dioxygenase-1 (IDO-1) is an enzyme in the kynurenine pathway which augments tumor-induced immune tolerance. Previous studies in childhood acute myeloid leukemia (AML) have shown a negative correlation of IDO-1 mRNA expression with outcomes. The aim of our study was to develop a practical and objective immunohistochemical technique to quantify IDO-1 expression on diagnostic bone marrow biopsies of AML patients in order to facilitate its use in routine clinical practice. IDO-1 mRNA was extracted from diagnostic bone marrow specimens from 29 AML patients. IDO-1 protein expression was assessed in 40 cases via immunohistochemistry and quantified by a novel ‘composite IDO-1 score’. In a univariate analysis, higher age (p = 0.0018), male gender (p = 0.019), high risk cytogenetics (p = 0.002), higher IDO-1 mRNA (p = 0.005), higher composite IDO-1 score (p < 0.0001) and not undergoing allogeneic stem cell transplant (SCT, p = 0.0005) predicted poor overall survival. In a multivariate model that included the aforementioned variables, higher composite IDO-1 score (p = 0.007) and not undergoing allogeneic SCT (p = 0.007) was found to significantly predict poor outcomes. Further, patients who failed induction had higher composite IDO-1 score (p = 0.01). In conclusion, ‘composite IDO-1 score’ is a prognostic tool that can help identify a certain subset of AML patients with ‘early mortality’. This unique subset of patients can potentially benefit from specific IDO-1 inhibitor therapy, currently in clinical trials.

## Introduction

Indoleamine 2,3 dioxygenase (IDO-1), an enzyme in the kynurenine pathway, plays a critical role in tumor-mediated immune tolerance^1^. It achieves this by catabolizing tryptophan, and by producing immunomodulatory kynurenine^2^. In addition to this, IDO-1 has been shown to have a non-enzymatic function as a ‘signaling protein’ within plasmacytoid dendritic cells^3^. The importance of this enzyme in normal host immunomodulatory processes is illustrated by the fact despite evolutionary forces; it is highly conserved in several animals, fungi and bacteria^4^. The potential role of IDO-1 in acquired immune tolerance was first suggested when inhibition of IDO in pregnant mice caused spontaneous immune rejection of allogeneic fetuses^5^. Since then, this immunomodulatory function of IDO-1 has been at least partially linked to disease progression and pathogenesis of certain chronic infections^6,7^, transplantation^8,9^, autoimmune diseases^10,11^ and malignancies such as breast carcinoma^12,13^, endometrial carcinoma^14^, serous ovarian tumors, melanoma^15^, hepatocellular carcinoma^16^ and colonic adenocarcinoma^17,18^. In tumors, inhibition of the IDO pathway is theorized to help ameliorate a state of immune privilege created by tumor cells enhancing endogenous T-cell mediated response against the tumor^17^. In the case of Acute Myeloid Leukemia (AML), preclinical studies in both adults and children have found a positive correlation of increased expression of IDO1 mRNA or functional activity in leukemic blasts correlated with worse overall survival (OS)^19–21^. This has prompted initiation of a clinical trial in which the IDO-pathway inhibitor indoximod will be combined with standard idarubicin/cytarabine chemotherapy in newly-diagnosed adult AML (NCT02835729). In adults, the subset of patients with by far the worst prognosis, fails to enter remission with induction chemotherapy. These patients often have a relentlessly downhill course despite best available therapy, and in certain high-risk populations such as elderly patients, over half will be dead within 6 months of diagnosis. This “early-mortality” subset represents a population that is in particularly urgent need of improved treatment. We hypothesized that the early-mortality population might represent patients with the highest IDO-1 expression, and thus the candidates most in need of an IDO-inhibitor drug as a component of their treatment regimen. At present, however, there are only a few clinical scores to predict in advance which patients will fail induction^22^ and it would be useful to have other novel biomarkers such as IDO-1 to predict induction success or failure. We hypothesized that immunohistochemical staining of initial diagnostic bone-marrows biopsies for a combination of extent and intensity of IDO-1 staining might be used to generate an objective pathologic score of IDO-1 expression; and that this would allow accurate prospective identification of those patients at highest risk of induction-failure and early mortality.

## Results

### Clinical characteristics

Data from forty patients was included in the final analysis. Median age at diagnosis was 60 years (range: 27–89); with 16 males (40%); and 22 self-reporting Caucasian (55%). Cytogenetic and molecular risk stratification included good in 3 patients (7.5%), intermediate in 32 patients (80%) and poor in 5 patients (12.5%). The French-American-British (FAB) classification distribution included M1 6 (15%), M2 5 (12.5%), M3 1 (2.5%), M4 5 (12.5%), M5 8 (20%), M6 2 (5%) and AML secondary to MDS 11 (27.5%). Twenty-nine patients (72.5%) underwent standard anthracycline and cytarabine induction, while 4 (10%) were treated with hypomethylating agents, and 7 (17.5%) untreated or treatment status unknown. Six patients (15%) underwent allogeneic stem cell transplant (SCT) and all of them were performed at the time of first complete remission (CR1). Twenty patients (50%) achieved remission, and among those 10 (25%) had subsequent relapse. Median overall survival (OS) was 283 days (range: 32–1941); with 8 alive (20%) at the time of data analysis. Table 1 provides a summary of patient and disease characteristics.

**Table 1:** Table showing baseline patient characteristics of forty AML patients included in the study, stratified by low (<0.45) and high (≥0.45) composite IDO-1 score (The cut-off point of 0.45 was derived after a ROC analysis).

**Patient characteristics (n** **=** **40)**	**Number of patients (%)**	**Composite IDO-1 score** **<** **0.45 (%)**	**Composite IDO-1 score ≥0.45 (%)**	**Median censored OS (in days)**	**P value**
Age					
<55	13 (32.5)	9 (69.2)	4 (30.8)	747	0.02*
56–74	19 (47.5)	5 (26.3)	14 (73.7)	284	0.6
>75	5 (12.5)	1 (20)	4 (80)	155	0.06
Sex					
Male	16 (40)	3 (18.8)	13 (81.2)	217	0.02*
Female	24 (60)	12 (50)	12 (50)	553	
Race					
Caucasian	22 (55)	6 (27.3)	16 (72.7)	317	0.07
African- American	18 (45)	9 (50)	9 (50)	331	
FAB subtype					
M0	0 (0)	0 (0)	0 (0)	—	—
M1	6 (15)	1 (16.7)	5 (83.3)	340	0.5
M2	5 (12.5)	1 (20)	4 (80)	282	0.3
M3	1 (2.5)	1 (100)	0 (0)	—	0.08
M4	5 (12.5)	4 (80)	1 (20)	—	0.001*
M5	8 (20)	4 (50)	4 (50)	665	0.5
M6	2 (5)	0 (0)	2 (100)	151	0.17
M7	0 (0)	0 (0)	0 (0)	—	—
AML with myelodysplasia-related changes	11 (27.5)	2 (18.2)	9 (81.8)	207	0.005*
FAB subtype unknown	2 (5)	2 (100)	0 (0)	—	0.4
Risk category (based on cytogenetics/mutational analysis)					
Good	4 (7.5)	3 (75)	1 (25)	—	
Intermediate	27 (80)	12 (44.4)	15 (55.6)	377	
Poor	9 (12.5)	0 (0)	9 (100)	147	0.002*
Allogeneic stem cell transplant					
Yes	6 (15)	4 (66.7)	2 (33.3)	—	0.0005*
No	30 (75)	10 (33.3)	20 (66.7)	217	
Unknown	4 (10)	1 (25)	3 (75)	711	
Treatment					
Standard induction ± maintenance	29 (72.5)	13 (44.8)	16 (55.2)	500	0.07
Hypomethylating agents	4 (10)	1 (25)	3 (75)	253	
Untreated/unknown	7 (17.5)	1 (14.3)	6 (85.7)	207	
Remission					
Yes	20 (50)	13 (65)	7 (35)	756	**<** 0.0001*
No	19 (47.5)	1 (5.3)	18 (94.7)	147	
Unknown	1 (2.5)	1 (100)	0 (0)	—	
Relapse (If remission was achieved)					
Yes	10 (25)	6 (60)	4 (40)	625	<0.0001*
No	10 (25)	7 (70)	3 (30)	—	

### IDO-1 mRNA has a negative correlation with OS

**Figure 1:**
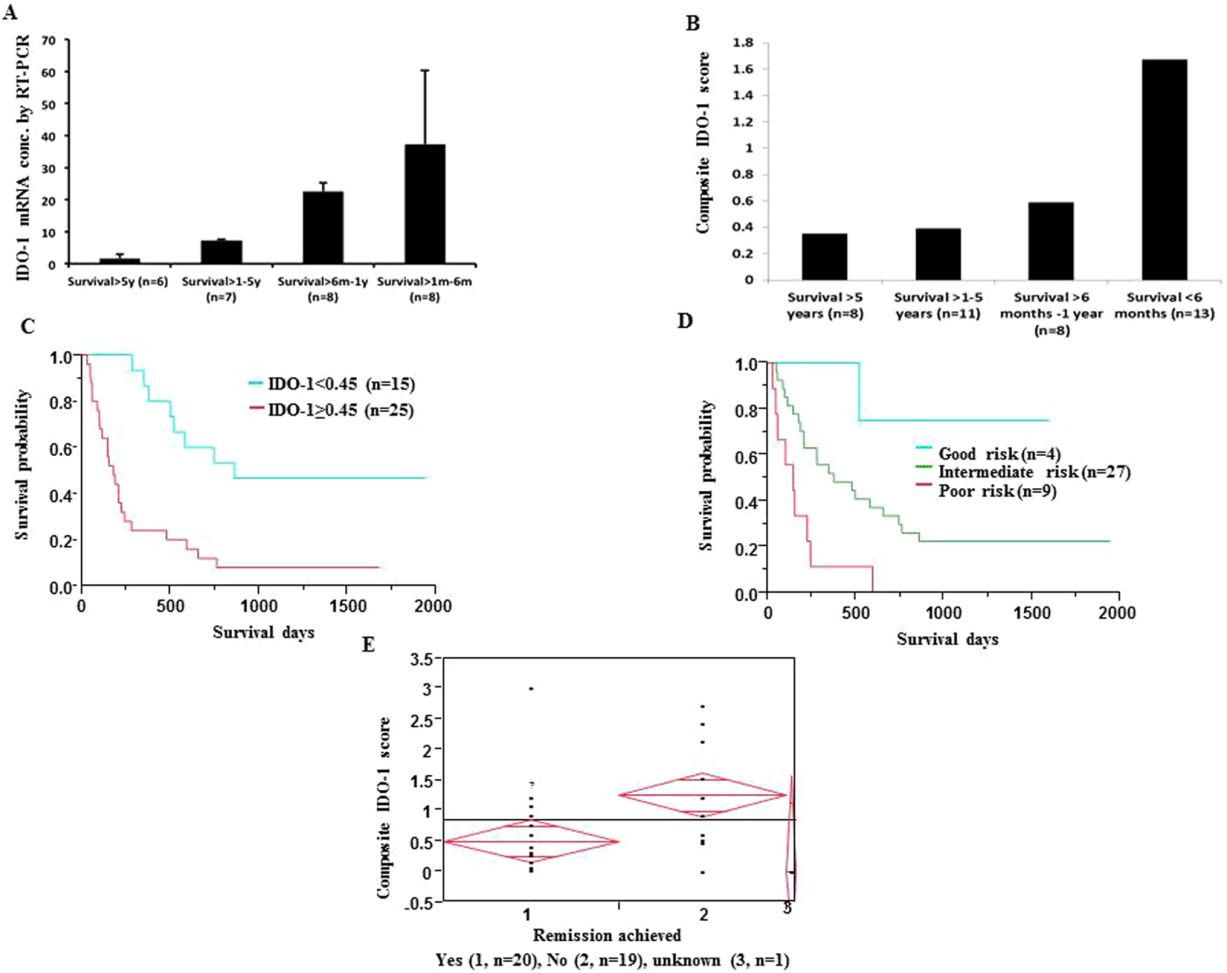
Composite IDO-1 score at diagnosis identifies patients at the highest risk of induction failure and early death. (**A**) IDO-1 mRNA expression by RT-PCR, normalized to 18S ribosomal RNA. The highest IDO-1 expression was found in the shortest survival group. Increased IDO-1 mRNA correlates with a shorter OS (*p = 0.01). (**B**) Composite IDO-1 score for each of the 4 survival groups, with highest IDO-1 expression correlating with a shorter OS. (**C**) Kaplan-Meir survival analysis of patients arbitrarily divided into two cohorts based on their composite IDO-1 score: “low” (<0.45) and “high” (≥0.45) (*p = 0.0005). (**D**) Kaplan-Meir survival analysis of composite IDO-1 score among the three risk groups as per the ELN classification system (*p = 0.005). (**E**) Figure showing patients who failed induction had higher composite IDO-1 scores (*p = 0.01).

IDO-1 mRNA expression (n = 29) was compared across the 4 different survival groups defined in Methods (Fig. 1A), with the highest levels in <6 month group (“early-mortality” group). Values were measured as per the fold change, with 18S as the housekeeping gene. 1.39, 0.99, 0.36 and 0.31 were the respective fold change values in groups 1, 2, 3 and 4 respectively.

### IDO-1 protein expression has a negative correlation with OS

Analysis of IDO-1 protein expression by immunohistochemistry (IHC) (n = 40) also showed a similar statistical correlation. After a receiver operating characteristic (ROC) analysis, a cut-off point of composite IDO-1 score was arrived at 0.45 and samples were divided into two groups: high (≥0.45) and low (<0.45). Kaplan-Meir survival analysis highlights direct correlation of poor survival with higher composite IDO-1 score (p = 0.0005, Fig. 1B,C). When patients were stratified by conventional risk categories, the highest composite IDO-1 score was seen in the poor risk category (median 1.5, n = 9), followed by intermediate (median 0.6, n = 27) and good risk categories (median 0.13, n = 4, p = 0.005, Fig. 1D). Further, IDO-1 mRNA and protein expression had a positive correlation as assessed by one-way analysis of variance (ANOVA, p = 0.024).

### Multivariate analysis proves composite IDO-1 score to be an independent prognostic factor

In a univariate model that included age, sex, race, body-mass index (BMI), allogeneic SCT, induction treatment strategy (standard cytarabine and anthracycline induction versus hypomethylating agents), remission achieved versus not, diagnostic peripheral and bone marrow blast percentage, only higher age (p = 0.0018), male gender (p = 0.019), high risk cytogenetics (p = 0.002), higher IDO-1 mRNA levels (p = 0.005), higher IDO-1 protein expression (as measure by composite IDO-1 score, p < 0.0001) and not undergoing allogeneic stem cell transplant (SCT, p = 0.0005) all correlated with a poor OS. However in a multivariate model which included age, sex, allogeneic stem cell transplant, IDO-1 mRNA levels, composite IDO-1 score and risk category, only higher composite IDO-1 score (p = 0.003) and not undergoing allogeneic SCT (p = 0.007) remained significantly associated with poor OS (Table 2). Multivariate analysis was performed using the Cox proportional hazards model.

**Table 2:** Table describing multivariate analysis (MVA) of our cohort (n = 40). MVA included variables significant in univariate analysis as below.

**Variable**	**Hazard’s ratio**	**95% Confidence interval**	**P value**
Age at diagnosis	0.99	(0.94, 1.04)	0.64
Male gender	2.2	(0.09, 1.80)	0.27
Higher risk category	1.13	(0.88, 0.22)	0.28
IDO-1 mRNA fold change	0.71	(0.24, 1.91)	0.5
Composite IDO-1 score	5.6	(1.76, 19.94)	0.003*
Lack of Allogeneic SCT	23.7	(2.91, 533.2)	0.007*

### Induction failure was inversely correlated with composite IDO-1 score

One-way analysis of variance (ANOVA) of composite IDO-1 score was compared with remission achieved with either one or multiple rounds of induction therapy (either with standard cytarabine and anthracycline induction versus hypomethylating agents) and we found that patients who did not achieve remission had higher composite IDO-1 score (p = 0.01, Fig. 1E).

### The Cancer Genome Atlas (TCGA) data confirms the prognostic role of IDO-1

**Figure 2:**
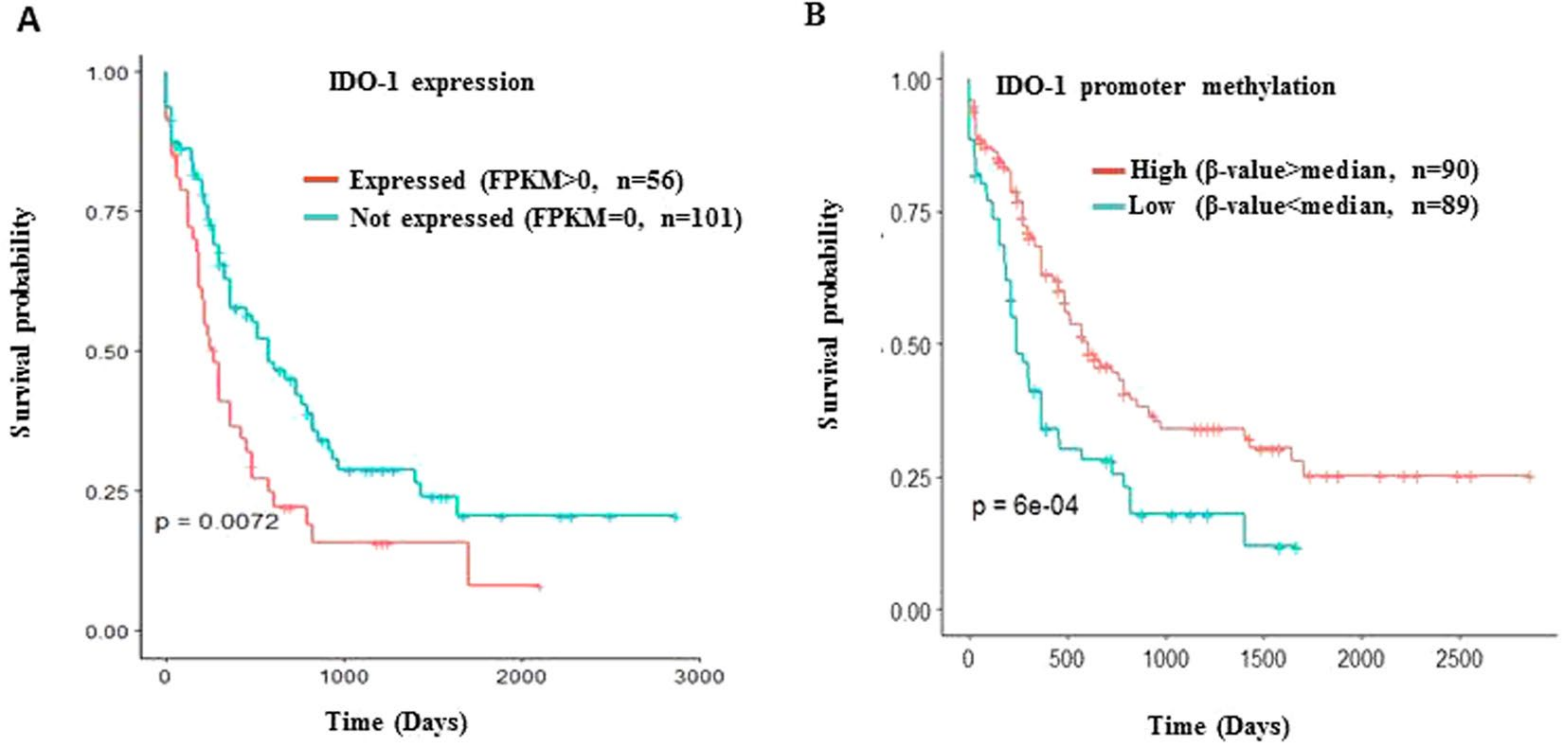
TCGA database of AML patients downloaded from the UCSC website. (**A**) RNA seq and Illumina 450 K methylation array data was obtained and the FKPM value was used to divide the external cohort into two groups for survival analysis. Higher FKPM >0 correlates with a poor OS (*p = 0.0072). (**B**) For methylation array data, one of the three CpG sites assigned to IDO-1 (*cg10262052) was used to divide the cohort into two groups based on the median β-value. Higher median β-values of IDO-1 promoter methylation correlated with better outcomes (*p = 6e-04).

The importance of IDO1 as a prospective risk classifier was validated from the external TCGA AML database. High β median value of IDO-1 promoter methylation status correlated with worse OS (p = 6e-04). Survival analysis of the TCGA AML patients’ cohort based on IDO1 mRNA expression is shown in Fig. 2A and promoter methylation is shown in Fig. 2B.

## Discussion

IDO-1 expression has been shown to affect survival in multiple tumors, including AML. Our study shows that increased IDO-1 mRNA and protein expression correlates with a lower OS. Importantly, increased IDO-1 expression in leukemic blasts at diagnosis, defined by a composite IDO-1 score performed on readily-available, archival diagnostic biopsies, correctly identified the subset of patients with highest risk of early mortality. In a multivariate model, high composite IDO-1 score at diagnosis was found to be an independent predictor of poor outcomes in AML.

In AML, tumor microenvironment is inherently hostile to immune effector cells^19^. IDO-1 is hypothesized to be a critical contributor to this phenomenon. Similar to our results, Chamuleau ME *et al*. showed that high IDO-1 mRNA in blasts was shown to correlate with poor outcomes^20^. Also, IDO-1 activity was shown to correlate with poor outcomes in children by Folgiero *et al*.^19^. Constitutive IDO-1 expression in AML blasts has been shown in about 50% of cases of AML, with the remaining half showing upregulation of IDO-1 mRNA after IFN-y stimulation^19,21^. Hara T *et al*. further confirmed the prognostic role of IDO-1 mRNA in combination serum L-kynurenine level^23^. Previous studies with allogeneic SCT suggest that immunologic mechanisms such as graft versus leukemia immunity play a critical role in clearance of leukemic blasts. Therefore, it seems reasonable that inhibition of a potent immunosuppressive mechanism such as IDO-1 might further enhance this immunotherapeutic effect and improve outcomes. The current study shows that it is possible to prospectively identify a subset of AML patients with the highest IDO-1 expression at the highest risk for early mortality. This may help as a companion diagnostic approach to identify those patients most likely to benefit from the addition of IDO-inhibitor drugs to front-line therapy for AML.

Despite the fact that we used rigorous statistical modeling and an external cohort to validate our results, there are certain limitations of our analysis. Due to small sample size, it is possible that our multivariate analysis is underpowered. To address this limitation, we used an external cohort (TCGA) to confirm our results. Due to fewer numbers and the fact that all patients with allogeneic SCT were performed at CR1, survival data for this variable should be interpreted with caution. Mutational analysis was not used as whole or targeted exome sequencing analysis was not performed on our cohort. It would be interesting to assess how IDO-1 expression correlates with different mutations.

## Materials and Methods

### Patient samples

Fresh-frozen paraffin embedded (FFPE) diagnostic bone marrow biopsy specimens were retrospectively collected from forty AML patients after obtaining institutional review board (IRB) approval. Detailed clinical, laboratory and outcomes data was recorded. Risk categories were defined as per the 2010 European Leukemia Net (ELN) classification system (due to limited numbers, intermediate-1 and 2 sub-categories were regrouped under one intermediate group)^24^. Messenger RNA (mRNA) was extracted from 29 specimens using a commercial kit (Qiagen) according to manufacturer’s protocol, following which, IDO-1 gene expression was checked via real time quantitative PCR. IDO-1 protein expression was checked via immunohistochemistry in 40 specimens. *All methods were performed in accordance with the relevant guidelines and regulations of Augusta University and were approved by its ethical committee and IRB. Prior bone marrow biopsy specimens were used and do not require informed consent*.

### Reagents

Mouse monoclonal anti human IDO antibody (#M256) purchased from CalBioReagents (San Mateo, CA, USA). ABC kit (PK-6102) was from Vector lab (Burlington, CA, USA). AEC-Substrate (HK092-5K) from Biogenex (Fremont, CA). Faramount aqueous mounting medium (S 3025) and ready to use Proteinase K solution (S 3020) from DAKO (Carpintena, CA) was utilized.

### RNA isolation and cDNA synthesis

For real time quantitation of IDO1 gene expression, total RNA was isolated from FFPE tissue blocks using Qiagen (QIAGEN Inc, Valencia, CA) mRNAeasy kit (217504) as per manufacturer’s protocol. DNase treatment step during isolation of total RNA eliminated the residual DNA carryover. The quantity of the total RNA samples was determined by ultraviolet spectrophotometer (Nanodrop, Thermo Scientific, and Pittsburgh, PA). Total RNA (200 ng) was reverse transcribed using iScript cDNA synthesis kit (170–8891) from BioRad laboratories (Hercules, CA).

### Real time PCR

IDO-1 gene expression was measured by quantitative real time PCR using SYBR green chemistry (SYBR Green Supermix, BIO-RAD laboratories Inc., Hercules, CA) on a BIO-RAD RT-PCR system (MyiQ optic Model). Data was analyzed by ∆-∆CT method using 18S ribosomal transcript as endogenous control. Transcript specific oligonucleotide primers for real time PCR were designed. Primers were purchased from Integrated DNA Technology, Iowa. Following were the IDO-1 primers used: 5′-TATGTGTGGGGCAAAGGT-3′ (forward primer) and 5′-CTTGGAGAGTTGGCAGTAAGG-3′ (reverse primer).

### Composite IDO-1 Score

IDO-1 protein expression was quantified through IHC. Two slides were prepared for each of the 40 specimens, one for hematoxylin/eosin staining and the other for staining with IDO-1 antibody. IHC experiments were carried out on 4% FFPE tissues. 4 µm in thickness sections were deparaffinized in xylene and hydrated in water. Endogenous peroxidase was blocked with 0.3% hydrogen peroxide in distilled water for 10 minute in room temperature (RT). Slides were rinsed with distilled water 3 times and then with Phosphate buffered saline with Tween 20 (PBST) for 5 minutes. Proteinase K pretreatment for 12 minutes at RT was done for antigen retrieval. Slides then washed one times with PBST and were blocked with 0.5% nonfat dry milk in PBST for 30 minutes at RT. For detection of IDO-1 protein expression, specimens were incubated overnight at +4° C with antimouse IDO-1 antibody in a 1:100 dilution. Visualization of bound antibodies was carried out using a streptavidin-biotin-peroxidase complex and 3-amino-9-ethylcarbazole as chromogen. Normal breast FFPE sections were used as a negative control.

**Figure 3:**
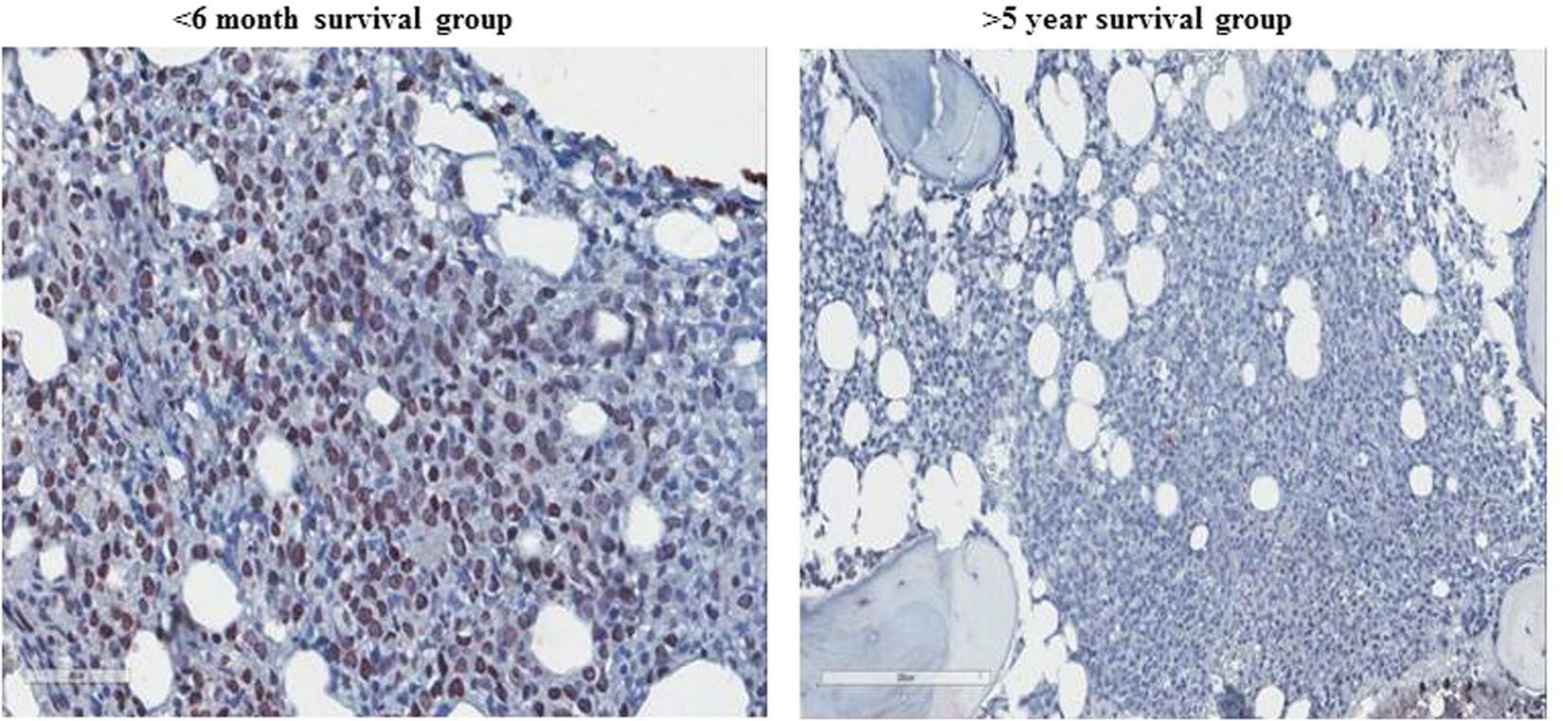
Representative example of IDO-1 staining by IHC in 2 outcome groups (<6 months and >5 year survival group) shown in the panel.

Percentage of mononuclear cells stained was noted and intensity of staining was graded as weak (1), moderate (2) or strong (3) by two independent pathologists (Fig. 3). Composite IDO-1 score was calculated by multiplying the grade of staining intensity by the percentage of stained mononuclear cells.

### Survival Groups

For analysis, patients were divided into 4 survival groups, defined as: >5 years (n = 8, group 1), 1–5 years (n = 11, group 2), 6 months-1 year (n = 8, group 3) and <6 months (n = 13, group 4).

### External cohort analysis

TCGA database of AML patients was downloaded from the University of California, Santa Cruz (UCSC) website. The RNA-seq and Illumina 450 K methylation array datasets are downloaded from the UCSC Cancer Genome Browser **(** https://genome-cancer.ucsc.edu **)**. The downloaded data are already processed and normalized by the UCSC Cancer Genome Browser team. The RNA-seq data are log2 transformed FPKM values. We did not perform any further processing on the downloaded RNA-seq data. The methylation data downloaded from the UCSC cancer genome browser are β-values subtracted by 0.5 and the values are between (−0.5 and 0.5). We restored the data to β-value by adding 0.5 to each data point. No other alteration was done to the methylation data set.

### Statistical analysis

Data was analyzed through JMP Pro (Version 7) statistical software. Unpaired t tests were used as appropriate. One-way ANOVA was used to assess correlation between variables. Multivariate analysis was performed using the Cox proportional hazards model. A p-value of 0.05 was considered significant and log-rank statistical test was used to derive the results. *Data is available for verification*.
